# An Immune Response to Heterologous ChAdOx1/BNT162b2 Vaccination against COVID-19: Evaluation of the anti-RBD Specific IgG Antibodies Titers and Interferon Gamma Release Assay (IGRA) Test Results

**DOI:** 10.3390/vaccines10091546

**Published:** 2022-09-16

**Authors:** Marzena Zalewska, Wiktoria Fus, Adam Konka, Karolina Wystyrk, Aneta Bochenek, Hanna Botor, Martyna Fronczek, Joanna Zembala-John, Brygida Adamek

**Affiliations:** 1 Department of Medical and Molecular Biology, Faculty of Medical Sciences in Zabrze, Medical University of Silesia in Katowice, H. Jordana 19, 41-808 Zabrze, Poland; 2 Silesian Park of Medical Technology Kardio-Med Silesia, M. Curie-Skłodowskiej 10C, 41-800 Zabrze, Poland; 3 Acellmed Ltd., M. Curie-Skłodowskiej 10C, 41-800 Zabrze, Poland; 4 Department of Pharmacology, Faculty of Medical Sciences in Zabrze, Medical University of Silesia in Katowice, H. Jordana 38, 41-808 Zabrze, Poland; 5 Department of Medicine and Environmental Epidemiology, Faculty of Medical Sciences in Zabrze, Medical University of Silesia in Katowice, H. Jordana 19, 41-808 Zabrze, Poland; 6 Silesian Center for Heart Diseases in Zabrze, M. Curie—Skłodowskiej 9, 41-800 Zabrze, Poland; 7 Department of Basic Medical Sciences, Faculty of Health Sciences in Bytom, Medical University of Silesia in Katowice, Piekarska 18, 41-902 Bytom, Poland

**Keywords:** COVID-19, SARS-CoV-2, immune response, vaccinations, T-cell immune response, immunoglobulin G, interferon-gamma release tests, humoral immunity, cellular immunity

## Abstract

This study aimed to assess the magnitude of anti-SARS-CoV-2 immunoglobulin G (IgG) titers and Interferon-Gamma Release Assay (IGRA) test results following administration of booster BNT162b2 in 48 ChAd-primed participants (vaccination schedule: ChAd/ChAd/BNT). Whole blood samples were collected: first, before and second, 21 days after the booster dose. The IgG level was measured using chemiluminescent immunoassay; the intensity of the T-cell response—IFNγ concentration—was assessed using IGRA test. At 21 days after the booster, all subjects achieved reactive/positive anti-SARS-CoV-2 IgG, and IGRA test results showed a significant increase compared to the results before booster administration. We compared the results before and after the booster between participants with and without prior history of COVID-19. The IFNγ concentrations in both cohorts were higher in convalescents (both before booster and 21 days after). The IgG titers were subtly lower in COVID-19 convalescents than in naïve but without statistical significance. Data on cell-mediated immunity are scarce, especially with regard to the general population. A better understanding of the complexity of the immune response to SARS-CoV-2 could contribute to developing more effective vaccination strategies.

## 1. Introduction

COVID-19 (Coronavirus Disease 2019) is a highly contagious illness caused by Severe Acute Respiratory Syndrome Coronavirus 2 (SARS-CoV-2), an unknown earlier pathogen belonging to the broad and diverse family of *Coronaviridae* [1,2]. It emerged at the end of 2019 in Wuhan, and within just a few weeks it spread throughout the world. Speed of transmission and its severe medical, social, and economic consequences led the World Health Organization (WHO) to the decision to pronounce, on 11 March 2020, COVID-19 a pandemic [3]. As of 8 September 2022, SARS-CoV-2 has contributed—according to the official data—to 603 711 760 confirmed cases and 6 484 136 deaths worldwide [4]. Today, over 2.5 years since the first case was reported in China, the COVID-19 pandemic is far from over. Moreover, its complex and long-term implications still constitute a great challenge for public health, the global economy, and politics [5].

Since the pandemic’s beginning, intensive research has been conducted—both on individual and population levels—on the changing SARS-CoV-2 molecular structure and properties of circulating and emerging variants in terms of their transmissibility, impact on immunity, and severity of infection they cause [6,7]. Simultaneously, numerous trials have been performed to understand different manifestations and courses of COVID-19 (depending on the variant that caused it) and find the most optimal methods of prevention and treatment [8,9].

A significant breakthrough in preventing the virus spread and altering the pandemic trajectory the world sought was achieved in the development and rollout of COVID-19 vaccines [10,11]. The first vaccines outside clinical trials were administered in the United Kingdom on 8 December 2020 [12]. The first products available on the market were based on using part of viral mRNA containing nucleoside-modified RNA (modRNA) in lipid nanoparticles, encoding the SARS-CoV-2 full-length spike glycoprotein (mRNA-1273, Moderna; BNT162b2, Pfizer/BioNTech; BNT). Another vaccine type available for the public at that time was based on the replication-deficient chimpanzee adenoviral vector, containing the SARS-CoV-2 structural surface glycoprotein antigen gene [7,13,14] (ChAdOx1-S (recombinant), the Oxford/AstraZeneca; ChAd) [15,16]. All products mentioned above were approved for use as a two-dose primary course.

Although vaccination is still considered the most effective defense strategy against SARS-CoV-2, multiple long-term follow-ups of vaccinated individuals conducted within clinical trials and real-world settings revealed that immune response to COVID-19 is waning over time [17,18,19]. Decreasing immunity has also been observed in individuals with COVID-19 history [20]. Moreover, numerous epidemiological studies report re-infections in vaccinated naïve subjects and both vaccinated and non-vaccinated convalescents [21,22]. In addition, a growing body of evidence indicates that particular population groups mount a limited or undetectable immune response to SARS-CoV-2 vaccines [23]. Low or non-responsiveness to COVID-19 inoculation can be related to, i.a., genetics, overall physical and mental health (i.a., stress), immune status, and presence of particular conditions (i.a., autoimmune and inflammatory diseases), such as advanced age and immunosenescence [23,24,25].

Those observations led to the introduction of a booster dose of the vaccine—to restore the protection against COVID-19-related serious outcomes. According to the current recommendations, it should be administered, depending on the product received during the initial series, optimally 4–6 months after completing the primary vaccination course [26,27,28]. Although the homologous strategy is still considered standard practice, due to changes in public health vaccination policy, and problems with vaccines’ availability, starting from Spring 2021, many countries decided to apply a heterologous booster [29,30]. Such an approach was initially documented as augmenting immune responses with tolerable reactogenicity [31,32,33].

The primary aim of active immunization with COVID-19 vaccines is to prevent severe disease, hospitalization, and death. Data on the vaccine-induced immune response and vaccines’ quality, safety, and efficacy have been robustly evaluated within clinical trials and numerous studies conducted before and after introducing the vaccines mentioned above into large-scale distribution [34,35]. It is also important to keep in mind that vaccines may have varying degrees of effects on new virus variants. Therefore, it is necessary to constantly update the content of vaccines and the body’s immune response to their effects [36]. In addition, FDA-approved drugs that minimize the effects of COVID-19 and reduce the number of hospitalizations are used with success [37]; however, it is vaccination that is attributed the greatest importance in the fight against SARS-CoV-2 infection [10].

Assessment of vaccine reactogenicity has focused, first and foremost, on measuring humoral and cellular response to SARS-CoV-2 spike following inoculation [38,39,40]. The first is evaluated based on the presence of specific immunoglobulin (Ig) directed against the spike (S) protein epitopes, primarily against the receptor-binding domain (RBD) of the S1 subunit of the SARS-CoV-2 spike protein (SARS-CoV-2 S1 RBD), using different serological antibody tests, i.a., chemiluminescent immunoassay (CLIA) [41]. It is worth noting that most studies investigating humoral response to SARS-CoV-2 concentrate on the measurement of immunoglobulins G (IgG), the most predominant class of immunoglobulins present in blood, and other human fluids. IgGs play an essential role in developing antibody-mediated immunity [42]. Meanwhile, cellular response is assessed by determining the presence of specific lymphocytes, i.e., Th1 CD4^+^ T and CD8^+^ T cells, using, e.g., ELISpot or flow cytometry [43]. Those methods, however, are costly, and their applications have certain limitations; therefore, they are not routinely used in SARS-CoV-2 diagnostics. An alternative approach to evaluating the intensity of the T-cell response is the measurement of interferon-gamma (IFNγ) concentration using Interferon-Gamma Release Assay (IGRA)—a tool already well established and successfully used, e.g., in the diagnostics of tuberculosis, introduced in a new version for SARS-CoV-2 research (SARS-CoV-2 IGRA version) [44]. IFNγ is a multipotent protein produced by lymphocytes (T cells) upon contact with specific pathogenic antigens—in our case, with SARS-CoV-2. Its presence is critical for developing innate and adaptive immunity, and, hence, the defense against COVID-19.

This prospective study aimed to assess the magnitude of the humoral response, expressed by anti-SARS-CoV-2 spike antigen-specific IgG, and the cellular response, expressed by the IFNγ level in the IGRA test, in adults following a booster dose (BNT) in ChAd-primed participants.

## 2. Materials and Methods

### 2.1. The Study Group

The study was conducted among individuals vaccinated with a two-dose regimen of ChAd and the BNT booster dose (ChAd/ChAd/BNT schedule) in the Silesian Park of Medical Technology Kardio-Med Silesia (Kardio-Med Silesia) in Zabrze. The primary vaccination course in this group was conducted between 2 March 2021 and 14 May 2021. The first dose was administered between 2–12 March 2021, and the second 4–12 weeks later (according to the WHO’s recommended immunization course). The booster dose in this group was given between 9 November 2021 and 29 December 2021.

The vaccination schedule discussed in this study was ChAd/ChAd/BNT. In addition, to evaluate the immune response to a booster dose, a measurement of 21 days post-booster was chosen, and study participants were monitored for possible SARS-CoV-2 infection for six months.

The inclusion criteria were: age ≥ 18 years old, being vaccinated with two doses of the ChAd in a primary course, with 8–12 week intervals between jabs, willingness to receive a booster dose of BNT, a medical permission to undergo vaccination with a third dose, approval to undergo two whole blood draws to measure the immune response before and after receiving a booster dose, accepting the study protocol and study schedule, and providing the informed consent to participate in the study (including follow-up period). Moreover, to prevent any deviations from the study protocol, only participants who received a two-dose regimen of ChAd in Kardio-Med Silesia and agreed to receive a booster dose at the same vaccination site could be included in the study.

**Figure 1:**
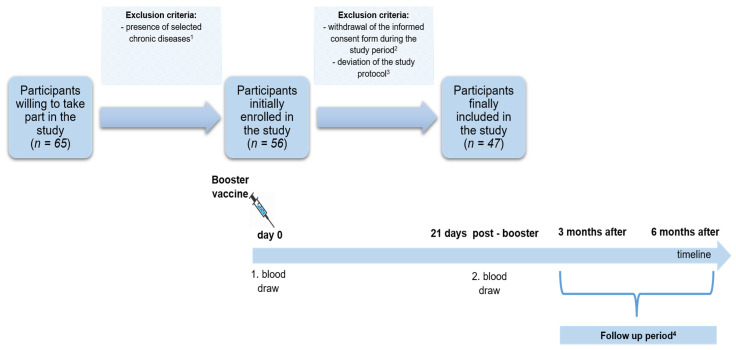
The study flowchart: study timeline and selection of the study group based on the exclusion criteria. Legend: ^1^ the presence of selected chronic diseases affecting immunoreactivity (i.e., autoimmune diseases, including Hashimoto’s disease, chronic obstructive pulmonary disease, diabetes); ^2^ withdrawal of the informed consent from the study (for personal or other reasons); ^3^ deviations of the study protocol (i.a., not being able to participate in the second blood collection 21 days after receiving the booster); ^4^ 6-month follow-up period after receiving a booster with potential occurrent SARS-CoV-2 infection (only RT-PCR confirmed) measured within three time intervals: <21 days (early follow-up, early FU), ≥21 days to 3 months (intermediate follow-up, intermediate FU), ≥3 to 6 months (late follow-up, late FU). Abbreviations: FU—follow-up.

Initially, 65 individuals declared their will to participate in our research. However, based on the survey performed at the study start, we decided to exclude patients with chronic diseases known to modulate immunoreactivity (i.e., autoimmune diseases including Hashimoto’s, chronic obstructive pulmonary disease, and diabetes) from further analyses. Finally, 47 subjects meeting the inclusion criteria were enrolled in the study (Figure 1).

Information on COVID-19 status before and after the booster dose was reported by respondents through the study authors’ survey and verified during telephone interviews.

Following the protocol, participants underwent two whole blood collections: first, at the study baseline, on the day of booster administration (before the injection), and second, 21 days after receiving a booster. In addition, all subjects were monitored for potential SARS-CoV-2 infection for up to six months following a booster within three time intervals for: (1) <21 days (early follow-up, early FU), (2) ≥21 days to 3 months (intermediate follow-up, intermediate FU), (3) ≥3 to 6 months (late follow-up, late FU) (Figure 1).

### 2.2. Laboratory Tests

During each blood draw, two aliquots of the whole blood were taken: one with a volume of 4 mL to a tube with a clot activator (Greiner Bio-One, Kremsmünster, Austria) for measurement of IgG antibodies and the second with a volume of 9 mL to a tube containing lithium heparin (Greiner Bio-One, Kremsmünster, Austria) for determination of IFN-γ concentration.

All analyses were conducted in the medical laboratory of the Silesian Park of Medical Technology Kardio-Med Silesia in Zabrze (Kardio-Med Silesia), accredited by the Polish Center of Accreditation (PCA). The study was conducted in accordance with the Helsinki Declaration. Furthermore, the study protocol was approved by the Bioethics Committee of the Medical University of Silesia in Katowice (No.: PCN/0022/KB1/50/II/20/21).

#### 2.2.1. Humoral Immune Response: Anti-SARS-CoV-2 IgG Measurement

The whole blood was centrifuged at a speed of 3500 rpm for 5 min to obtain serum samples. Humoral activity was tested using ACCESS SARS-CoV-2 (Beckman Coulter Inc., Brea, CA, USA)—a two-step enzyme chemiluminescent immunoassay (CLIA). It detects IgG antibodies at the receptor-binding domain (RBD) of the SARS-CoV-2 spike protein (S1). The selected test to identify anti-RBD of S1 protein IgGs had a lower limit of quantitation (LoQ) of 2.00 AU/mL (arbitrary units/mL) with an upper range possible to detect an IgG titer of 8000.00 (AU/mL). The test was performed according to the manufacturer’s instructions, and the outcomes were interpreted according to manufacturer’s guidelines [45]. The results were considered reactive when the IgG value was ≥10.00 AU/mL and non-reactive when the IgG value was <10.00 AU/mL.

#### 2.2.2. Cellular Immune Response: Quantitative Determination of IFN-γ Release by SARS-CoV-2-Specific T Cells

The Quan-T-Cell ELISA kit (EUROIMMUN Medizinische Labordiagnostika, Luebeck, Germany) was used to assess interferon-gamma (IFNγ) concentration according to the manufacturer’s instructions [46]. After gentle mixing, three heparinized fresh blood samples in the volume of 0.5 mL each were added to three different tubes included in the Quan-T-Cell kit and incubated at 37 °C for 20–24 h. The first tube did not contain any stimulating agent and was used to determine the individual IFNγ background; it was subtracted from the values obtained in the other two tubes. The second tube included the S1 domain of the SARS-CoV-2 spike protein. The third one, with a mitogen causing an unspecific IFNγ release, was used for controlling the stimulation ability. If stimulable immune cells were present in the sample, they were activated during the incubation to release IFNγ. After the incubation, the tubes were centrifuged at 12,000× *g* for 10 min to obtain stimulated heparinized plasma, which could be used to determine the IFNγ concentration. The values obtained in the three tubes were used to calculate the final concentration of IFNγ (mIU/m) released by T lymphocytes due to their stimulation with S1 protein. The results were based on a borderline range recommended by the manufacturer, where a value <100.00 mIU/mL was considered negative; 100.00–200.00 mIU/mL—borderline; and >200.00 mIU/mL—positive [46].

### 2.3. Statistical Analysis

Data were presented as counts and percentages for qualitative variables and as median, with first (Q1) and third (Q3) quartiles, for quantitative variables. The distribution of the data was evaluated using the Shapiro–Wilk test. Due to deviations from the normal distribution of the quantitative variables, non-parametric methods were implemented. The Wilcoxon test was applied to compare pairs of dependent variables and to assess the correlation between measurements over time with an additional grouping variable, and the nparLD test with a post hoc test of multiple comparisons was used.

The relationship between quantitative variables was analyzed using the Spearman correlation coefficient: *p* values <0.05 were considered significant. In the analyses, the R language in the Rstudio environment with the GGplot, npaLD, and one-way tests libraries were used [47,48].

## 3. Results

A total of 47 subjects met the inclusion criteria and were included in the study: 41 women (87.23%) and 6 men (12.77%). The mean age in this group was 47.00 years (range: 29.00–65.00 years, SD = 8.91). The study group’s basic demographic and clinical characteristics of the study group are presented below (Table 1).

In our study group, 13 participants (27.66%) underwent COVID-19 before the study started (i.e., before the booster administration); all infections were confirmed by a real-time Reverse Transcription Polymerase Chain Reaction (RT-PCR) test. In this cohort, almost all individuals (84.62%) had been infected before receiving the first dose of the ChAd vaccine. During the primary vaccination course, two subjects (15.38%) became ill after the first vaccine jab. According to the survey and respondents’ subjective opinions, none of the study participants experienced severe illness courses; moreover, none required hospitalization.

Performed tests revealed that 23 subjects (48.94%) of the study group before the booster administration had IgG antibody levels above 10.00 AU/mL (i.e., reactive), and only one person (2.13%) of the examined population had an IFNγ concentration below 100.00 mIU/mL (i.e., negative). The other 46 participants (97.87%) obtained IFNγ values above 200.00 mIU/mL, corresponding to a positive result. Interestingly, 7 out of 13 COVID-19 convalescents had IgG antibody levels <10.00 AU/mL (i.e., non-reactive), and 6 of them ≥10.00 AU/mL (i.e., reactive).

Twenty-one days after receiving a booster dose, all subjects (100.00%) achieved reactive/positive values of anti-SARS-CoV-2 IgG and IFNγ concentrations, i.e., above 10.00 AU/mL and above 200.00 mIU/mL, respectively (Table 2).

**Figure 2:**
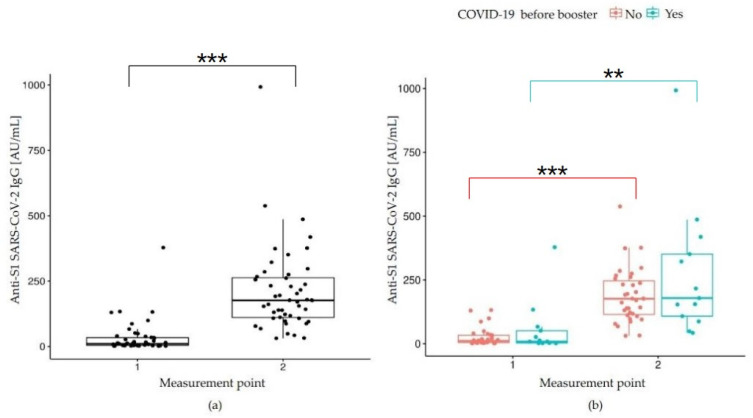
Anti-S1 RBD SARS-CoV-2 IgG (AU/mL) concentrations: (**a**) before a booster (1) and 21 days after (2), *p* < 0.001; (**b**) before a booster (1) and 21 days after (2) depending on the COVID-19 status before the study start. If a *p*-value is less than 0.01, it is flagged with 2 stars (**). If a *p*-value is less than 0.001, it is flagged with three stars (***).

Our study also investigated the dynamics of humoral and cellular responses to the BNT booster. Twenty-one days after administration of the booster injection, a significant increase in the IgG antibody level was observed (*p* < 0.001) (Table 2), indicating a moderate association between the IgG titer at the study baseline and after receiving a booster jab (r = 0.51, *p* < 0.05) (Figure 2a).

**Figure 3:**
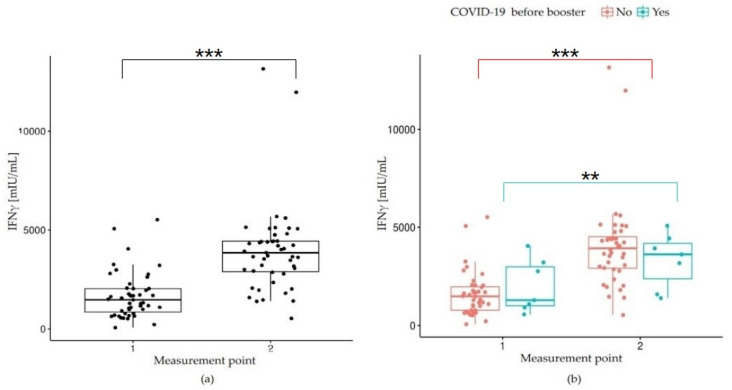
IFNγ concentration (mIU/mL) obtained in IGRA test: (**a**) before the booster (1) and 21 days after (2), *p* < 0.001; (**b**) before the booster (1) and 21 days after (2) depending on the COVID-19 history before the study start. If a *p*-value is less than 0.01, it is flagged with 2 stars (**). If a *p*-value is less than 0.001, it is flagged with three stars (***).

Similarly, twenty-one days after the booster administration, a significant increase in IFNγ concentration in the IGRA test was observed in the study population (*p* < 0.001) (Table 2 and Figure 3a). Spearman’s correlation analysis demonstrated a moderate correlation between the results obtained in two consecutive measurements (r = 0.49, *p* < 0.05).

To assess the impact of the booster dose and, in fact, of the fourth exposure to SARS-CoV-2 antigens on the humoral response, a comparison of IgG antibody levels before and twenty-one days after the booster dose was performed using an nparLD test from the nparLD R library (Figure 2b). Post hoc analysis showed a significant increase in IgG antibody levels 21 days after receiving the booster, both in the group of convalescents, i.e., with a history of COVID-19 before the recall dose (*p* = 0.002) and the COVID-19-naïve subjects (i.e., without COVID-19 history before the booster jab); (*p* < 0.001). We found no significance by comparing convalescents with naïve patients at each time point. Subtle differences between the groups indicated higher IgG antibody titers in naïve individuals. In contrast, 21 days after the third booster vaccine, the level of IgG antibodies was higher in COVID-19 convalescents than in naïve participants, but still this difference was not statistically significant (*p* > 0.05). The results of multiple comparisons (post hoc tests) are presented in Table 3 (Table 3).

Similarly, to evaluate the impact of the booster on the cellular response, IFNγ concentration levels before and 21 days after administration of the third vaccine dose were compared between two cohorts: participants with and without a prior positive history of COVID-19 before the study started. The comparison was performed using an nparLD test from the nparLD R library (Figure 3b). The post hoc analysis showed a significant increase in IFNγ concentration twenty-one days after the booster, both in the group of convalescents (*p* = 0.001) and participants who did not previously have COVID-19 (*p* < 0.001). Twenty-one days after administration of the booster dose: the concentration of IFNγ was higher in COVID-19 convalescents compared to naïve subjects, but with no significant effect (*p* = 0.348). The results of multiple comparisons (post hoc tests) are presented in Table 3.

In addition, we monitored our study group for six consecutive months. During the follow-up period, the SARS-CoV-2 infection was confirmed in seven (14.90%) individuals, all of them females. All became infected for the first time (and not due to breakthrough infection). In addition, one subject was diagnosed with COVID-19 during an early follow-up, six days after receiving the booster dose (Table 4).

Interestingly, all but one of the infected individuals had one or more conditions or risk factors known to affect the immune response [49,50,51,52,53]. Six subjects (85.71%) had an excessive body mass (BMI ≥ 25.00; mean: 29.53; range: 25.42–34.61), and two of them were obese (BMI ≥ 30.00). Moreover, two participants (28.57%), apart from being overweight or obese, had arterial hypertension. In addition, one overweight (14.29%) respondent was a long-term smoker (smoking ≥ 20 years).

All infections were test-confirmed (RT-PCR). There were no hospitalizations nor deaths in this group up to six months, neither from COVID-19 nor for other reasons (Table 4). It is worth mentioning that according to the official national data, at the time of booster administration and follow-up period, the most dominant variant of concern circulating in the area where the study was performed was Omicron (B.1.1.529) [54].

## 4. Discussion

Since the emergence of SARS-CoV-2, numerous studies have been conducted to understand the response of the immunological system to the novel infectious agent and its emerging variants, especially variants of concern (VoC).

The immunological system consists of two types of immunity: innate (natural) and adaptive (acquired), each built with different cell types and biologically active molecules. 

The adaptive, also called specific, immunological system consists of three major types of lymphocytes: B cells, producing antibodies, and T cells (CD4^+^ T and CD8^+^). It is activated as a result of the first exposure to a pathogen during infection or vaccination [55,56,57].

Two mechanisms of specific immunity are implemented to combat a given pathogen within the body: humoral (antibody-mediated) and cellular (T-cell-mediated) [55,56,57]. Those types of immune response collaborate closely with each other. Moreover, this multifaceted interplay provides recognition of an active pathogen and leads to the development of resistance to the same agent in the future. That is how the so-called “immunological memory” works [58,59,60,61,62,63,64,65,66].

Humoral response to a given pathogen is usually monitored on a timeline and evaluated based on the presence and quantity of antibodies (preferably neutralizing ones) in one’s blood. Most studies have investigated this type of response to SARS-CoV-2, as it is simpler and less expensive to measure than a cellular one. However, research protocols on anti-SARS-CoV-2 antibodies, as well as methods of measurement, vary considerably from one study to another, making the results difficult to compare [67,68,69,70].

In our study, we applied the enzyme chemiluminescent immunoassay to detect the anti- SARS-CoV-2 S1 RBD IgG antibodies. IgGs target the so-called “spike protein” of the novel coronavirus—it is against this epitope that both ChAd and BNT vaccine-induced immunity is directed.

Our research revealed that at the study baseline, i.e., before receiving a booster dose and over six months after completing the primary vaccination course with the ChAdOx1 vaccine, less than 50% of the study group maintained reactive concentration levels of IgG (≥10 AU/mL). Interestingly, in this group, there were only 6 out of 13 COVID-19 convalescents. Those observations go in line with other studies on the waning of humoral response over time, reported regardless of one’s COVID-19 status or type of vaccine administered [50,71,72,73]. Similar findings were reported by Daković Rode et al. in their research on anti-SARS-CoV-2 Spike IgG antibody dynamics after administration of two doses of the BNT vaccine [74]. A significant decrease in anti-SARS-CoV-2 IgG antibody levels (compared to 1-month follow-up results) was recorded as early as 3 months (3-month follow-up), both in COVID-19 convalescents (median 4831.00 AU/mL; *p* < 0.0001) and infection-naïve individuals (median 2976.70 AU/mL; *p* < 0.001). Moreover, six months after completing a primary vaccination course, a further significant decline in IgG titers was reported compared to the values obtained at the 3-month follow-up. This phenomenon was observed in both groups: with and without previous history of COVID-19 [74]. Shrotri et al., who investigated a humoral response in a group vaccinated with two doses of ChaAdOx1, reported that over two months after completing the primary vaccination course, the anti-S1 IgGs titer decreased about five-fold compared to the values obtained during the first weeks following a second dose [75]. In addition, results of the large study conducted in Israel by Levin et al. confirmed a significant waning of anti-S IgGs titers within six months following primary vaccination with the BNT, with a relatively stable rate in time [17].

A growing body of evidence on the rapid decline of anti-SARS-CoV-2 IgG antibody levels and possible decrease in vaccines’ effectiveness resulted in introducing recommendations for administering a booster dose [20,76]. After implementing the national boosting strategies and rollout of booster shots in many countries, a significant growth in antibody titers was documented, mainly within the several consecutive weeks following the booster jab. Jeong et al. analyzed this phenomenon in a group of health care workers (HCW) one month after administration of the BNT booster administration, following homologous prime vaccination with two doses of the ChAd vaccine. The results indicated a significant increase in median antibody titers compared to those obtained after the second dose [77]. Similar results were reported in our study group twenty-one days after booster administration.

Our study group consisted of participants who were not particularly exposed to coronavirus infection (e.g., healthcare workers); thus, we may assume that the risk of infection in this group reflected the risk of the local general population and, similarly, that their immune responses to the booster following the homologous primary regimen represented immunity of the local general population. It is worth mentioning that during our study, i.e., during booster administration and follow-up period, the most dominant variant of the SARS-CoV-2 virus in the region where our research was conducted was Omicron (aka B. 1.1.529, BA.X)—a VoC more transmissible than previous strains, known for its ability to largely evade immunity gained from past infection or two doses of vaccine [78,79]. It may partially explain the fact of the seven (14.90%) cases of SARS-CoV-2 infection, confirmed in our ChAd-primed group 4–12 weeks after receiving a booster jab.

When comparing the groups of participants who contracted COVID-19 before the booster dose and those who were naïve, we found no significant effect of additional exposure to the SARS-CoV-2 virus antigen on IgG antibody titers. The difference in median values between the group of participants who contracted COVID-19 before administration of the booster and the group of participants who did not contract seems to be subtle, although higher for the group of participants with no COVID-19 infection. We observed certain expected results also found in other studies; for example, in the research of Chang et al., the anti-RBD IgG analyses in vaccinated (previously positive for COVID-19) participants showed that these individuals had a significantly greater immune response against SARS-CoV-2. Their medians of IgG antibody titers were significantly higher than in naïve patients who received two or three doses of the vaccine [80]. In another study by Demonbreun et al., it was shown that after two doses of the vaccine (in this case the vaccine of BioNTech, Pfizer and Moderna) IgG antibodies were higher in individuals with a previous COVID-19 positive result compared to naïve participants [81]. However, it should be noted that the above works were carried out on a different vaccination schedule and with other vaccines. In addition, exposure to the SARS-CoV-2 viral load can be unequal. In our study, mainly changes in IgG antibody titers and the production of IFNγ before and after the administration of the booster dose were assessed. In addition, our study group was relatively small—reducing the study group by more than 25.00% compared to the baseline group (47 vs. 65 participants)—which was intended to eliminate the possible impact of chronic and autoimmune diseases on the immune response to vaccination. Therefore, it seems necessary to enlarge the study group to verify the results obtained.

The cellular response to SARS-CoV-2 has been relatively understudied, although there is growing evidence that most infected individuals develop robust and long-lasting T-cell immunity [82]. It is assumed that it is primary and, therefore, more effective and longer-lasting than a humoral response (represented as IgG titers), persistent even when pathogen cells escape antibody-mediated immunity [83]. In addition, it plays a critical role in performing rapid virus clearance [84]. According to numerous studies, in 20.00–30.00% of infected asymptomatic individuals or those experiencing mild symptoms, specific SARS-CoV-2 antibody levels remain below a detection threshold. In addition, as mentioned earlier, some people do not mount sufficient antibody concentrations through vaccination. Therefore, immune response after SARS-CoV-2 infection or vaccination can only be confirmed in those subjects by measuring cell-mediated immunity [85].

Based on the observation of IFNγ, producing T-cell responses to SARS-CoV-2 immunization through natural infection or vaccination, the IGRA was introduced as an attractive alternative method of cellular immunity assessment [86]. This approach is proven to be a valuable tool in determining SARS-CoV-2 immunity, as it relatively fast and provides high sensitivity and specificity [57].

In our study group, all but 1 (2.13%) participant had a positive IGRA test result before receiving a booster, which indicates that 46 individuals (97.87%) maintained a high potential for producing IFNγ concentration for more than six months after completing the primary series. This observation goes in line with recent findings. As mentioned earlier, Seraceni et al. reported high IFNγ concentration levels in their study participants after eight months from the second dose [86]. Furthermore, twenty-one days after the booster, positive values of IFNγ concentration were detected in all our study participants (100.00%). Similar results were reported by Jeong et al. in their study on IFNγ concentration in vaccines primed with two doses of ChAd: followed by BNT injection, one month after the booster, a 92.80% positivity was observed [77].

SARS-CoV-2 is unusually effective in its escape strategy by suppressing the body’s innate immune response, i.e., secretion of type I and type III interferons (IFNs) [55,87,88,89,90]. Delayed IFN induction and limited IFN signaling block an effective defense against SARS-CoV-2. Numerous studies confirmed that hindered production of IFN is strongly associated with failure to control a primary SARS-CoV-2 infection and a high risk of fatal COVID-19 outcomes [62,91,92,93,94,95,96,97,98].

In this study, we found no significant differences in IFNγ levels in the group that did not develop COVID-19 compared to the group that developed COVID-19 before the booster dose, as well as in the short period (21 days) after the vaccination booster. However, higher medians were visible in the convalescents. We did, however, find a significant increase in IFN-gamma after booster doses in both groups: those who were not diagnosed with COVID-19 and those with the infection. Such an increase after vaccination was also observed in other works with differences between vaccination schedules. For comparison, in a study conducted for 243 people, in which patients were divided into groups (individuals who had previously received two doses of BNT162b2, individuals who had received vaccinations with BNT162b2 + ChAdOx1, individuals who were double vaccinated with ChAdOx1), prior to the booster dose (BNT162b2), the group that was vaccinated with the BNT162b2 + BNT162b2 schedule had lower levels of IFNγ than the group vaccinated with the ChAdOx1 + ChAdOx1 schedule. The study showed the highest increase in INFγ release in subjects with the ChAdOx1 + ChAdOx1 + BNT162b2 vaccination schedule. However, these participants showed the weakest humoral (IgG) response prior to the booster dose [99].

Adaptive immune responses and immune memory are crucial for effective vaccination results. Booster vaccination and a heterologous three dose schedule proved to induce the immune system more effectively than homologous regimens [57].

Recognition of different aspects of adaptive immune response to coronavirus infection and vaccination against COVID-19 and a better understanding of factors affecting one’s immunity on the timeline is critical in combating SARS-CoV-2 successfully. Unfortunately, despite almost two years since introducing anti-SARS-CoV-2 vaccines into real-world settings, the level of our knowledge on the long-term vaccine effectiveness remains insufficient. In addition, the protective threshold of antibody and cell-mediated response to SARS-CoV-2 is yet to be determined. Establishing an optimal and convenient methodology allowing for reliable estimation and comparison of adaptive immunity parameters could significantly facilitate and improve the research.

## 5. Conclusions

The immune response to SARS-CoV-2 is a very complex phenomenon, dependent not only on antibody-mediated and cell-mediated mechanisms but also on many other host- and pathogen-related factors. Therefore, higher levels of anti-SARS-CoV-2 antibodies, stimulated T-cell interferon-gamma production, or even both cannot be considered—unambiguously and definitely—as determinants of sufficient protection or being more susceptible to contracting COVID-19.

Moreover, the immune response following boosting might also depend on the history of COVID-19 infection and infection-induced immunity. In our study, the humoral response (measured by IgG antibody titer) after the booster dose was similar in both the vaccinated convalescents and vaccinated naïve groups.

The cellular immune response expressed by IFN-gamma levels in the IGRA test was apparently higher in the vaccinated convalescents. Therefore, it can be concluded that a hybrid immune response to SARS-CoV-2, i.e., infection- and vaccine-induced, seems superior to that developed through infection or vaccination.

Further research is required to understand primary vaccine-induced longitudinal immunity to SARS-CoV-2.

## 6. Study limitations

Our study has some limitations. Due to implemented inclusion and exclusion criteria and the risk of participants’ failure to comply with the study protocol, our group was relatively small. In addition, there was a predominance of females, which might skew the study results. Moreover, due to the nature of SARS-CoV-2 infection caused by the Omicron variant, the most dominant VoC circulating in the study region during the follow-up period, and the similarity of its symptoms to the common cold, thus, the risk of potential error in self-reported diagnosis, we decided to include only information on RT-PCR-confirmed infection. In the light of emerging variants and subvariants, evading post-vaccinal immunity, research on the humoral and cellular immunity following vaccination and boosting is crucial and cannot be underestimated.

## Figures and Tables

**Table 1:** Basic demographic and clinical characteristics of the studied cohort.

Variable		*n*	%
Sex	male	6	12.77
	female	41	87.23
Age (years)	<60	42	89.36
	≥60	5	10.64
BMI	underweight (<18.50)	1	2.13
	normal body mass (18.50–24.99)	30	63.82
	overweight (25.00–29.99)	10	21.28
	obesity (≥30.00)	6	12.77
Smoking	yes	6	12.77
	no	41	87.23
COVID-19 before a booster dose	yes	13	27.66
	no	34	72.34
COVID-19 after a booster dose	yes	7	14.89
	no	40	85.11

Abbreviations: BMI—body mass index.

**Table 2:** Anti-SARS-CoV-2 IgG (CLIA-measured) and IFNγ concentrations (IGRA-measured) in the homologous (ChAd/ChAd) recipients before and after receiving a BNT booster.

Parameter	Range	*n* (%) 47 (100.00%)	Mean ± SD	Median (Q1; Q3)	*p*-Value
IgG before a booster (AU/mL)	<10.00	24 (51.06)	33.06 ± 61.90	10.38 (4.35; 34.34)	*p* < 0.001
	≥10.00	23 (48.94)			
IgG after a booster (AU/mL)	<10.00	0 (0.00)	211.41 ± 162.96	176.65 (110.00; 264.45)	
	≥10.00	47 (100.00)			
IFNγ before a booster (mIU/mL)	<100.00	1 (2.13)	1664.16 ± 1158.19	1472.43 (777.76; 2037.33)	*p* < 0.001
	100.00–200.00	0 (0.00)			
	>200.00	46 (97.87)			
IFNγ after a booster (mIU/mL)	<100.00	0 (0.00)	3975.47 ± 2198.65	3890.92 (2897.18; 4601.09)	
	100.00–200.00	0 (0.00)			
	>200.00	47 (100.00)			

Legend: IgG ≥ 10 AU/mL—reactive; IgG < 10 AU/mL—non-reactive; IFNγ < 100 mlU/mL—negative; IFNγ 100–200 mlU/mL—borderline; IFNγ ≥ 200 mlU/mL—positive. Abbreviations: IgG—immunoglobulin G, IFNγ—interferon-gamma, SD—standard deviation, *p*—statistical significance.

**Table 3:** Changes in the concentration of IgG antibodies and interferon-gamma concentration levels in the study group (*n* = 47) depended on SARS-CoV-2 infection in anamnesis before and after receiving a BNT booster. Results of multiple comparisons (post hoc tests).

		SARS-CoV-2 Infection before a Booster Dose		
		Yes *n* = 13 (27.66%)	No *n* = 34 (72.34%)	Yes Vs. No
Parameter	Estimation point	Median (Q1; Q3)	Median (Q1; Q3)	*p*-value **
IgG (AU/mL)	Before a booster (1)	8.62 (3.05; 51.25)	11.22 (4.50; 33.81)	*p* > 0.05
	After a booster (2)	179.05 (108.22; 351.25)	176.40 (111.77; 255.47)	*p* > 0.05
	*p*-value *	*p* = 0.002	*p* < 0.001	
IFNγ (mIU/mL)	Before a booster (1)	1673.92 (1093.17; 2622.30)	1291.26 (696.22; 1951.83)	*p > 0.05*
	After a booster (2)	4414.46 (4231.84; 5095.95)	3540.81 (2347.16; 4426.45)	*p* = 0.348
	*p*-value *	*p* = 0.001	*p* < 0.001	

Legend: * post hoc test *p*-value for comparison of IgG and IFNγ concentration levels before (1) and after (2) booster administration in the group of the same COVID-19 status: either cohort of convalescents (yes) or COVID-19 naïve (no) before the study started; ** post hoc test *p*-value for comparison of IgG and IFNγ concentration between two groups: convalescents (yes) and COVID-19 naïve (no) before the study started in two estimation points, i.e., before (1) and after (2) receiving a booster. Abbreviations: IgG—immunoglobulin G, IFNγ—interferon-gamma, Q1—first quartile, Q3—third quartile, *p*—statistical significance.

**Table 4:** Results of the 6-month follow-up period: diagnosed SARS-CoV-2 infection (RT-PCR-confirmed) in the study group (primary ChAd/ChAd recipients), following a (BNT) booster administration (*n* = 47).

	Follow-Up Period		
Follow-Up Outcomes	Early FU (<21 Days)	Intermediate FU (≥21 Days–3 Months)	Late FU (≥3–6 Months)
SARS-CoV-2 infection *n* (%)	1 (2.13)	6 (12.77)	0 (0.00)
death *n* (%)	0 (0.00)	0 (0.00)	0 (0.00)

Abbreviations: FU—follow-up.

## Data Availability

The data used to support the findings of this research are available from the corresponding authors upon request.
